# Dr. Fisher’s Reply to an Editorial in the “Texas Medical Journal”

**Published:** 1897-12

**Authors:** 


### DR. FISHER'S REPLY TO AN EDITORIAL IN THE "TEXAS MEDICAL JOURNAL."

I feel it a duty that I owe my brother physicians, as well as myself, to answer an editorial that appeared in the November issue of the TEXAS MEDICAL JOURNAL, edited and controlled by Dr. F. E. Daniel, paid secretary of the State Health Officer. To leave it unanswered would perhaps serve the apparent purpose intended by the author to create a wrong impression in the minds of his readers and bolster up the inconsistent position that his superior officer has taken and maintained with great consistency from beginning to end.

We will take his editorial by section. In speaking of Dr. Guiteras he says:

First—"We feel a sense of shame and humiliation that he should have received at the hands of the medical profession of

Galveston so little consideration; if indeed the action of a large element of the profession at that place, subsequent to the doctor's visit, were not indeed an indignity."

In the name of justice and the real facts in the case, what did the medical profession of Galveston do to justify such an accusation? I challenge Dr. F. E. Daniel, or any one else, to mention one instance in which Dr. Guiteras was treated discourteously or spoken of disrespectfully by a single Galveston doctor; such a statement is without foundation in fact. The physicians of Galveston, to a man, considered Dr. Guiteras, a gentleman, a scholar, and a man of unusual care and research, and considered him a man who deserved all the editor said, perhaps more. I again challenge the editor to name the doctor who cast insinuations, "as that he went to Galveston in the interest of New Orleans trade." Mr. Editor, sustain your position or retract. I ask the profession and all intelligent people; Is it a crime to respectfully disagree with a brother physician? Simply because the Lord has endowed you with a conscience and courage of your convictions? This is the only shadow of an indignity that was shown Dr. Guiteras (if such it can be considered). There were many in our midst who were close observers and who had had the experience of several yellow fever epidemics, and because they disagreed with Dr. Guiteras, they are the cause of "shame and humiliation." Well, if this be grounds for "shame and humiliation" you can put me in that class.

Second. "There is a little unwritten history connected with the visit of Dr. Guiteras to Galveston, which we feel inclined to ventilate for the purpose of throwing some light upon the insistence of Surgeon General of M. H. S., that there was yellow fever in Galveston long anterior to the coming of Dr. Guiteras, and as showing the reason why Dr. Guiteras was sent there," etc.

I think the whole unwritten history told in a straight forward manner would be of more interest to our readers than the "little unwritten history" of the editor.

My reasons for declaring Dr. Swearingen's quarantine "a perfect farce" are plainly stated in an interview published in the Galveston News, of Sept. 23, 1897, headed: "Dr. Fisher on the Stand." I have no apology to offer for the position taken at that time regarding the State quarantine, but have witnessed such an amount of laxity in State quarantine matters, that I consider my position had not only been sustained, but absolutely strengthened.

Now for the Sealy hospital case. That, according to the editor, was "more than suspicious." On the 10th of September, there was admitted to the hospital a Mexican by the name of Guadalupe Torres, with a history that he had been sick six or seven days prior to his admission, without medical aid. Dr. H. P. Cooke, a physician, careful, capable and conscientious, treated this patient and diagnosed his case catarrhal jaundice. On the second or third day after admission to the hospital, Dr. Cooke left the city; during his absence, on the fourth day after his admission, the man died. An autopsy was made by Dr. Allen J. Smith, assisted by Dr. Starley and one of the internes. The autopsy was incomplete; only the stomach, liver and spleen was examined; no examination of kidneys, heart or brain. Dr. Smith sent for me, told me of his finding, and was fearful he had a case of yellow fever. I asked him the ground for his apprehension, and his reply was that he relied on what the autopsy revealed, etc. I did not agree with him, because the personal history of the individual showed no exposure. The clinical history certainly did not point to yellow fever, and the autopsy was so incomplete that it would not be possible for any pathologist to sustain his position on the findings. Dr. Smith suggested that I should report this case. This I would not do, because I did not think I had anything that would justify me in exciting our citizens without rhyme or reason. To have reported this case would have been a complete stultification of pathologists, health physician, and all connected with it. There never was any cases of even a suspicious character as a result of this case, and many were exposed to it. On the very evening of the autopsy, I noted the arrival of Dr. R. M. Swearingen in the city (in the evening paper), but he never came near me, or any member of the Board of Health, nor did he visit the hospital. After Dr. Smith had informed me of the autopsy, I tried, by telephone, every hotel in the city, to locate Dr. Swearingen, to secure his personal inspection of the case; he was not found at any of them. I then telephoned Dr. Mayfield, at the State Quarantine Station, and he then informed me that Dr. Swearingen had left on the evening train. Witnesses to the above were Dr. Allen J. Smith, Dr. Starley, and the superintendent of the John Sealy Hospital.

Again the editor says: "The State Health Officer never heard of the occurrence of this case until thirteen days after the death and autopsy." Is this statement correct? Let us examine the facts.

I here introduce a letter from Dr. Starley, and telegram from Dr. Swearingen, with my answer thereto, and date of each:

THE JOHN SEALY HOSPITAL,
NINTH AND STRAND.

W. F. STARLEY, M. D.,

SUPERINTENDENT AND HOUSE SURGEON.

GALVESTON, Nov. 18, 1897.

*Dr. W. C. Fisher, Galveston.*

DEAR SIR:—The Mexican, Guadalupe Torres, was admitted to the John Sealy Hospital September 10th, 1897, and died September 14th, 1897. An autopsy was performed on the day of death. Very truly yours,

W. F. STARLEY, JR.,
Superintendent.

(TELEGRAM).

AUSTIN, Texas, Sept. 17, 1897.

*Dr. W. C. Fisher, Health Officer, Galveston, Texas.*

What of the Mexican, Guadalupe Torres, who recently died at the City Hospital. Did Dr. Smith or any one hold autopsy, if yes, with what results. R. M. SWEARINGEN,

(TELEGRAM).

GALVESTON, Sept. 17, 1897.

*Dr. R. M. Swearingen, Austin.*

From clinical history, personal history as to locality catarrhal jaundice, now thirteen days since inception of disease, no developements suspicious. F. K. FISHER."

(My message was given to the telegraph operator over telephone and he signed F. K. Fisher, who is my brother, and who was at that time on a Mallory S. S. bound for New York. This was simply a clerical error on the part of the operator or the receiver of the message). According to my calculation fourteen and three are seventeen. Dr. Swearingen's telegram to me was on the 17th., and the autopsy according to Dr. Starley's letter was on the 14th. Mr. Editor, am I not correct in asserting that your statement is incorrect? There is an insinuation in the editorial that a health officer should be tarred and feathered who would conceal a more than suspicious case; what should be done with an editor who handles the truth so recklessly? Even at the risk of injuring the name of a fellow citizen, I say not only that he should be tarred and feathered, but ridden on a rail.

Does the editor consider Dr. Allen J. Smith (a man who by his own word of mouth admitted on the night of the autopsy, that he had never seen a case of yellow fever, nor had he held an autopsy on a yellow fever case) a competent authority? Would he be classed an expert; would his opinion be superior or equal to that of men who had treated many hundreds of cases of yellow fever and had made many autopsies; and when I told them of his 'ncomplete investigation and his findings therein, they smiled *almost audibly*. I have no apologies to offer for my course in this case, either to the State health officer, or his subordinate, the editor, and in a similar case and under similar circumstances my course would be the same. The results in this case I think proved beyond a doubt, the wisdom of my course, and the correctness of my views. The autopsy to the contrary notwithstanding. I wish it distinctly understood that what I have said regarding Dr. Smith, applies, of course, to *yellow fever*. I have the highest regard for him as a teacher, pathologist and gentleman. There was hardly any reason in my replying to Dr. Swearingen's telegram regarding the Torres case as to Dr. Smith and the autopsy. He had the name and the facts so pat that I supposed that he had some kind friend in the city who either from fright or officiousness desired to apprise Dr. Swearingen of this *remarkable case*. Information concerning this case doubtless went to Washington and other places, indeed and on good authority, I heard that it was even published in the Philadelphia paper. Who gave Dr. Swearingen his information, is a matter of little concern to me. I would have given it to him in person, had I been able to find him when he was in the city, and after he left I never considered it of sufficient importance. We are able to manage cases of catarrhal jaundice without the assistance of the State health officer. I do not know that under the circumstances, it was incumbent upon me to reply to telegrams of the State health officer. I have on several occasions wired to him but for reasons *best known to him* my telegrams have been unanswered.

I will have to have better authority for the statement than the Torres case was the reason that the Surgeon-General sent an expert to Galveston, than that of the editor. We have shown that Dr. Swearingen was informed of this case on September

17th, and evidently knew of all the facts. By reference to the Galveston News on the 24th of September, I notice in an Austin dipatch that Surgeon Wyman sent a telegram to Secretary Daniel of the State Health Department that he had received a wire that there were cases of yellow fever in Galveston and San Antonio and desired to know whether the Health Officer at Austin had any such information. Dr. Daniel replied that they knew nothing of any case at either place, or words to that effect. This dispatch you will notice was from Austin September 24th. This hardly tallies with the statement that Surgeon-General Wyman in some way got this information and telegraphed Dr. Swearingen. "I have information by wire seemingly perfectly reliable that yellow fever exists in Galveston." Now, if Dr. Swearingen regarded our case of "catarrhal jaundice" yellow fever and had information of same on September 17th (as was shown by his telegram to me and my answer to him), why could he not enlighten Wyman on the 24th. It ought not to take any man in matters of such importance to deliberate seven days.

It is about as unreasonable an assertion to give this case as the reason for Dr Guiteras coming here as it was to admit freight from Algiers into Texas and telling the people it was safe and the State had a rigid quarantine. I would now like to call attention to this quotation: "Now, when 'suspicious cases' (?) occur in Galveston; nay, when the highest authority in both the National and State service pronounced it yellow fever, and quarantine is declared against Galveston; there are none so loud in their protests as Dr. Fisher." Well, this is a dandy! The editor must have had a case of jim-jams or be suffering from some form of mental aberration. Two days prior to the announcement of Dr. Guiteras, that we had eight cases of yellow fever I was seized with an attack of dengue; my diagnosis and that of my attending physician, Dr. Trueheart, though Dr. Guiteras diagnosed my case yellow fever—in order that we will show favors to none we will call it Denguiteras; and for any person suffering with this disease "to be loud in his protest" is simply too ridiculous, too absurd for anything. The idea of of a doctor writing such a thing, granting that he never had a case himself, had never treated a case, he ought to know better from professional literature. I had a temperature of 104 F. when Dr. Guiteras made the announcement of yellow fever, and was not able to be out to attend to business for two weeks thereafter; and Mr. Editor still says "there were none so loud in their protest as Dr. Fisher." He also speaks of the highest authority in the State service pronouncing it yellow fever and declaring quarantine. This is one of the most wonderful strides of inconsistency that the State has been guilty of. We presume our Health Officer is familliar with all matters pertaining to yellow fever, and quarantine—the incubative stage, etc.

On Tuesday following Dr. Guiteras' report, the State Health Officer announces that both Galveston and Houston are infected towns, in other words, have yellow fever, and declared quarantine against them. On the following Friday he telephoned a merchant of this city "if the Board of Health would declare no yellow fever, I will raise quarantine." And after the board declared there was no fever, which they could do with consistency, which they had never declared there was, on Saturday following, three days, he raised quarentine; who ever heard of such a procedure. It was the crowning inconsistency of a most inconsistent management of State quarantine affairs. I append the following telegram sent from my sick bed on Friday evening, the day before the raising of quarantine by the State Health Office:

GALVESTON, Oct. 16th, 1897.

*Dr. R. M. Swearengen, State Health Officer, Austin, Texas.*

You quarantined Galveston on the report of Dr. Guiteras and on your personal responsibility, and not on a report of the Board of Health of the city of Galveston, for it has never reported yellow fever; you alone hold the key to the situation. Do us the justice to examine our patients, and every facility will be afforded you, and for God's sake relieve us of this useless quarantine. Answer when we may expect you.

W. C. FISHER,
Health Physician.

My purpose in sending this telegram was to keep myself properly on record in regard to quarantine matters. I received no reply thereto as had happened so often before.

W. C. FISHER, M. D.,
Health Physician, City of Galveston.

